# Generalized-stacking-fault energy and twin-boundary energy of hexagonal close-packed Au: A first-principles calculation

**DOI:** 10.1038/srep10213

**Published:** 2015-05-22

**Authors:** Cheng Wang, Huiyuan Wang, Tianlong Huang, Xuena Xue, Feng Qiu, Qichuan Jiang

**Affiliations:** 1 Key Laboratory of Automobile Materials of Ministry of Education & School of Materials Science and Engineering, Nanling Campus, Jilin University, No. 5988 Renmin Street, Changchun 130025, PR China

## Abstract

Although solid Au is usually most stable as a face-centered cubic (*fcc*) structure, pure hexagonal close-packed (*hcp*) Au has been successfully fabricated recently. However, the phase stability and mechanical property of this new material are unclear, which may restrict its further applications. Here we present the evidence that *hcp* → *fcc* phase transformation can proceed easily in Au by first-principles calculations. The extremely low generalized-stacking-fault (GSF) energy in the basal slip system implies a great tendency to form basal stacking faults, which opens the door to phase transformation from *hcp* to *fcc*. Moreover, the Au lattice extends slightly within the superficial layers due to the self-assembly of alkanethiolate species on *hcp* Au (0001) surface, which may also contribute to the *hcp* → *fcc* phase transformation. Compared with *hcp* Mg, the GSF energies for non-basal slip systems and the twin-boundary (TB) energies for 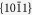 and 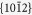 twins are larger in *hcp* Au, which indicates the more difficulty in generating non-basal stacking faults and twins. The findings provide new insights for understanding the nature of the *hcp* → *fcc* phase transformation and guide the experiments of fabricating and developing materials with new structures.

In recent years, many new materials of nanoparticles have been synthesized and applied in potential domains. Note that there is often phase transformation among face-centered cubic (*fcc*), body-centered cubic (*bcc*) and hexagonal closed-packed (*hcp*) systems in mono-component nanocrystal superlattices, especially for the noble metal nanostructures^1^,^2^,^3^,^4^,^5^,^6^,^7^,^8^,^9^. This can be attributed to the diffusion of guest molecules (e.g. dodecanethiol)^3^, surface stress^4^,^5^, specific stacking processes depending on evaporation kinetics^6^ and high pressure^7^,^8^ etc. Solid Au is usually most stable as an *fcc* structure. Surprisingly, Huang *et al.*^1^ have reported the first *in situ* synthesis of pure *hcp* Au square sheets on graphene oxide, which possess an edge length of 200–500 nm and a thickness of ~2.4 nm (~16 Au atomic layers). The exclusive *hcp* phase, which is stable under ambient conditions, provides a significant basis for fabricating novel Au architectures with unique chemical and physical properties^10^. Recently, it is observed that a large value of initial residual stress drives the *fcc*/*hcp* phase transformation in [100]/{100} Au nanowires during the energy minimization process, as conducted by molecular dynamics simulation^5^. Moreover, Stoeva *et al.*^11^ reported that Au particles obtained by the solvated metal atom dispersion method predominantly organized into *hcp* nanocrystal superlattices with long-range translational ordering.

Note that structural defects such as point defects, stacking faults, dislocations, twins and grain boundaries can be observed in nanocrystal superlattices^12^, which are similar to the phenomena shown in classical crystals. In *hcp* Au nanocrystal, the stacking faults and twin defects were both indicated by transmission electron microscope (TEM)^1^. With the appearance of faulted stacking in *hcp* structures, the ideal *hcp* packing sequence (…*ABABAB*…) would exhibit partial *fcc* feature, e.g. the *ABC* sequence in I_2_ (…*ABABCACA*…) and T_2_ (…*ABABCBABA*…) stacking faults. Therefore, the stacking faults in one system can be actually regarded as the presence of a segment of the other^2^. Moreover, the mixed stacking of *hcp* and *fcc* planes was confirmed in Au plates, suggesting that the pure *hcp* phase will become less stable when the Au sheets grow thicker^1^. Since the close relationship between the stacking faults and phase transformation, it is necessary to investigate the defect properties of *hcp* Au, so as to evaluate the structural stability and growth mechanism of this new material.

Furthermore, the first synthesis of *hcp* Au stimulates us to explore its novel mechanical properties and to make comparisons with common *hcp* structures, e.g. Mg, in anticipation of developing new materials which are distinct from those natural crystals for wider application. On the atomic level, the plastic formability of *hcp* crystals is closely related to the ease of the formation of dislocations and twins. Note that the GSF energy, which quantifies the ability of the dislocation in a crystal to glide onto an intersecting slip plane, affects the nucleation and mobility of dislocations^13^ as well as the propensities to form twins^14^. Meanwhile, the TB energy is intimately connected with the mobility of twinning dislocations^15^, without referring to the atomic shuffling during the nucleation of twins in *hcp* crystals. Therefore, the TB energies mainly depict the stability of twin structures without involving the formation dynamics.

Though these defect parameters are significant in reflecting the mechanical properties of *hcp* Au, they are difficult to be investigated experimentally due to the structural instability. Moreover, a universal *fcc* to *hcp* phase transformation is possible only when the dimension of Au nanostructure decreases to a critical value, which also increases the difficulty in detecting the *hcp* Au phase by experiments. Alternatively, the first-principles method based on density functional theory (DFT)^16^ has emerged as a key technology in determining the properties of material, which enables the calculation of defect energies at a reasonable computational cost. Therefore, the main purpose of this paper is to study the energies of stacking faults and twin boundaries in *hcp* Au using first-principles method. For the calculation of GSF energy, the basal slip system (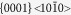 and 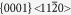), prismatic slip system (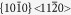) and pyramidal slip system (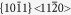 and 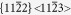) are considered respectively, as illustrated in Fig. 1. Meanwhile, the 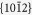 mirror reflection, the 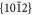 mirror glide as well as the 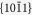 mirror reflection and the 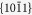 mirror glide twin boundaries are taken into account when calculating the TB energy. In addition, the elastic properties of *hcp* Au, which are associated with the brittleness and ductility of materials^17^, are also calculated for the systematic study of *hcp* Au. For comparison, the corresponding physical parameters for *fcc* Au and *hcp* Mg are listed as well, in order to estimate the structural stability and application of *hcp* Au. Note that a lot of surfactant molecules adsorb on the surface of Au nanostructure during the synthesis processes^1^,^2^,^3^. Therefore, the effect of the thiol surfactant to the crystal lattice of *hcp* Au is also investigated. The intrinsic parameters in this calculation contribute to completing the theoretical database of *hcp* Au, and act as a guide to experiments of fabricating and developing materials with new structure.

## Results and discussion

### Structural parameters

The calculated equilibrium lattice constants and cohesive energy for *fcc* Au, *hcp* Au and Mg are listed in Table 1. For *hcp* Au, the structural parameters (a = 2.952 Å, c = 4.885 Å) in our work are in acceptable tolerance with the values of the experiment (a = 2.96 Å, c = 4.84 Å)^1^ and other simulation (a = 2.927 Å, c = 4.903 Å)^18^. The *c/a* ratio of *hcp* Au (1.655) is larger than that of Mg (1.608), implying larger anisotropy in lattice parameters for *hcp* Au. Note that the lattice constant for primitive cell of *fcc* Au (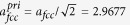 Å) is very close to that of *hcp* Au (2.952 Å), which reveals the intrinsic link between the *fcc* and *hcp* phase. Moreover, the cohesive energy (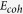) of *hcp* Au (−3.2045 eV) is negative, indicating that the existence of this new phase is energetically favorable. According to the energetic results, the stability of crystal is sequenced as: *fcc* Au >*hcp* Au.

### Surfactant effect

In *hcp* Au nanostructure, a lot of surfactant molecules adsorbed on its surface^1^,^2^. In view of the molecule-surface interactions, the effect of the surfactant, e.g. thiols, to the lattice constant of *hcp* Au is investigated. Spontaneous formation of an ordered molecular over-layer on the gold surfaces has been found for the thiols, namely molecular self-assembly. A series of experiments has revealed the Au (111) surface releases gold adatoms that become incorporated into the monolayer^19^,^20^,^21^,^22^. This configuration is further confirmed by the Au top-site adsorption of alkanethiolate in experiments^23^,^24^.

For *hcp* Au (0001) surface, the Au-adtom-induced self assembly of alkanethiolate species is presented analogy to the *fcc* Au (111) surface. Accordingly, a 4 × 2 slab (along the 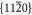 and 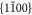 direction) with 9 layers was constructed, separated by a 12 Å vacuum. Upon relaxation, the Au adatom locates at the twofold bridge site and the headgroup-S atom of CH_3_S occupies the Au top-site as shown in Fig. 2a,b. The distribution of Au adatom and alkanethiolate species on *hcp* Au (0001) surface is similar to that on *fcc* Au (111). This originates from the same in-plane atomic coordination on the two close packed planes. Meanwhile, the S atom is attached to both the Au adatom (Au^a^ in Fig. 2a) and the underlying lattice atom (Au^l^ in Fig. 2a) with r(S-Au^a^) = 2.344 Å and r(S-Au^l^) = 2.523 Å respectively. These values are very close to the 2.33 Å and 2.49 Å in the case of adoption of alkanethiolate on *fcc* Au (111)^19^. The binding energy of the CH_3_S- species and the Au substrate is 2.08 eV, which is smaller than the value of cohesive energy for *hcp* Au, i.e. 3.2045 eV (Table 1).

Note that the formation of bare *hcp* Au (0001) surface is accompanied by charge transfer from the dangling bonds into the in-plane bonds, which increases the attraction between the surface atoms. The adoption of S with strong electronegativity leads to a charge redistribution into the surface-molecule bonds, which weakens the attraction between surface Au atoms and increases the superficial lattice constant. For the adoption of alkanethiolate on *hcp* Au (0001) surface, the average in-plane distance between Au^l^ and surrounding Au atoms (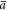) is increased from 2.952 Å to 2.997 Å. Such adsorbate-induced surface stress has also been confirmed in alkanethiolate-Au (111) self-assembled monolayers^25^. Moreover, the adsorption of alkanethiolate surfactant results in increased tensile stress when the S-C bond is normal to the surface, and therefore gives rise to a significant expansion of the Au lattice (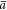: 3.049 Å). This situation is remedied to some extent by the tilting of the S-C bond (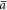: 2.997 Å). The resulting directionality of the S-C bond leads to a preferred value of the C-S-Au^l^ bond angle of 106.5°.

The covalent character of the S-Au bond also expands the inter-planar spacing between the superficial *hcp* Au (0001) planes slightly. The average distance between the A1 and A2 layers (see Fig. 2a, 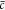) is 4.895 Å for the adsorption of alkanethiolate species, which is 4.890 Å on the bare *hcp* Au (0001) surface. Note that the titling of the S-C bond also alleviates the expansion of inter-planar spacing, i.e. the value of 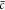 is 4.912 Å if the S-C bond is normal to the surface. The lattice parameters in the underlying layers (a = 2.952 Å, c = 4.887 Å) are similar to those of *hcp* Au bulk (a = 2.952 Å, c = 4.885 Å).

The adsorption of alkanethiolate molecules on *hcp* Au (0001) surface enlarges the gold lattice in the superficial layers (e.g. the three outer layers), while it has slight effect in the underlying layers. The extension of surface lattice constants may facilitate the *hcp*  Æ  *fcc* phase transformation, as the lattice parameter a of *hcp* Au (2.952 Å) is smaller than that for the primitive cell of *fcc* Au (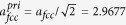 Å). Moreover, the *fcc* Æ*hcp* transformation of Au is proven to involve the compression of lattice along the 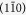 direction^22^, and it in turn is predicted to be an expansion process for the *hcp* *→* *fcc* transformation. However, more experimental evidences are in expectation.

### GSF energy

**Figure 1:**
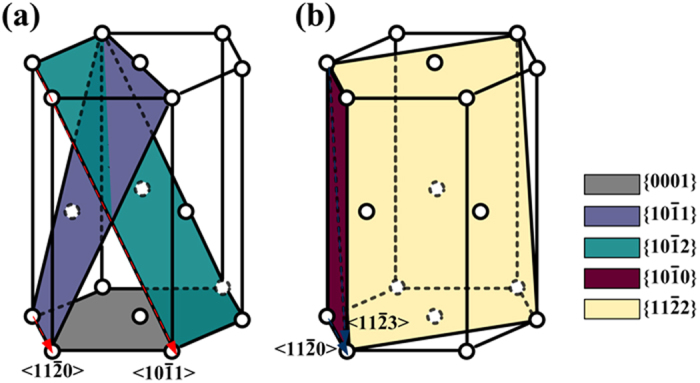
Schematic representation of different slip systems for *hcp* crystals: (**a**) 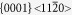, 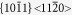 and 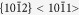 slip systems; (**b**) 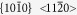 and 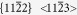 slip systems.

**Figure 2:**
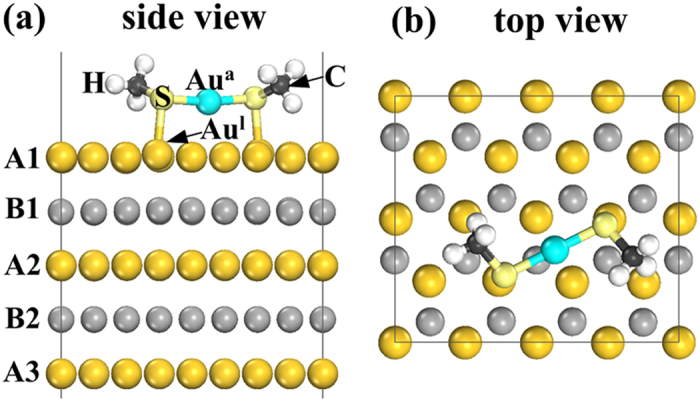
Schematic illustrations of the Au-adtom-induced self-assembly of alkanethiolate species on *hcp* Au (0001) surface: (**a**) side view (5 layers are shown) and (**b**) top view (2 layers are shown).

To calculate the GSF energies of *hcp* Au, which reflects its bulk property, a 16-layer slab (32-atom) is constructed based on the optimized unit cell (a = 2.952 Å, c = 4.885 Å). Five slip systems for both *hcp* Au and Mg are taken into account, as shown in Fig. 1. Specifically, the three < a > -type slip systems (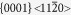, 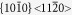 and 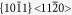) are considered. Moreover, the second pyramidal slip system (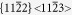), which is responsible for accommodating the strains of **c**- and **a**-axis simultaneously, is also calculated. Meanwhile, the basal slip system (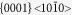) is considered, as the dissociation of perfect dislocations into partial dislocations is energetically favorable^26^:


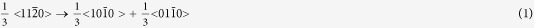


For *fcc* Au, the GSF energies are calculated in the primary slip system (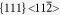).

**Figure 3:**
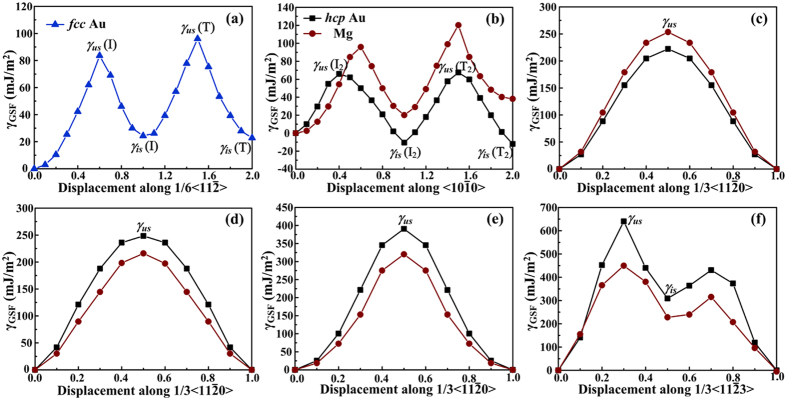
GSF energy curves for *fcc* Au in (**a**) 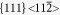 slip system; for *hcp* Au and Mg in (**b**) 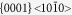, (**c**) 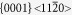, (**d**) 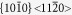, (**e**) 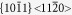 and (**f**) 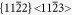 slip systems.

The GSF energy curves (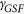-curve), which express the initiation of one whole stacking fault, were plotted against the applied shift vector for *fcc* Au, *hcp* Au and Mg in Fig. 3a–f. Hereinto, the maximum energy on the 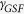-curve is the unstable SFE 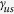, which denotes the lowest energy barrier for dislocation nucleation^27^, while the local minimum indicates the intrinsic SFE 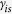
28. The computed 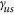 and 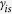 values are marked on the 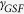 curves and summarized in Table 2, together with those obtained by other first-principles predictions^29^,^30^,^31^,^32^,^33^,^34^. Note that the GSF energies for Mg_32_ in different slip systems accord with the results calculated by others^29^,^30^,^31^,^32^,^33^,^34^, which proves the accuracy of our calculations.

In slip systems 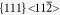 (Fig. 3a), 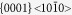 (Fig. 3b) and 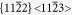 (Fig. 3f), the 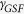 curves keep saddle-shaped, in which the local maximum and minimum values both exist. However, there is no local minimum on the GSF energy curves of the 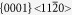 (Fig. 3c), 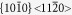 (Fig. 3d) and 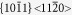 (Fig. 3e) system.

In 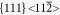 system of *fcc* Au, the 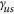 (83.6 mJ/m^2^) and 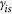 (24.3 mJ/m^2^) values for the intrinsic I stacking fault are close to the results (94 and 27 mJ/m^2^) of Wu *et al*^32^. For some common *fcc* metals, the energy barrier (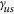) for the initiation of the I structure is: 169 mJ/m^2^ (Al)^35^, 180 mJ/m^2^ (Cu)^35^, 305 mJ/m^2^ (Ni)^36^, 111 mJ/m^2^ (Ag)^32^, 311 mJ/m^2^ (Pt)^32^ and 215 mJ/m^2^ (Pd)^32^. By comparison, the relatively low GSF energy for *fcc* Au indicates the ease of slip and thus, good plasticity.

In *hcp* Au and Mg, the 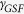 curves for different slip systems show similar trends in variation, e.g. the existence of 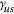 and 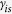 corresponding to approximately the same fault vector (abnormal: 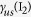 in 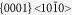 system). The GSF energies of *hcp* Au for five slip systems are sequenced as: 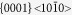 < 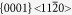 < 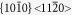 < 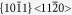 < 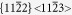.

In the basal 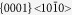 slip system, the 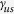 and 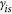 values are apparently lower for *hcp* Au than those for *hcp* Mg, and are even lower than those for *fcc* Au. Specially, the 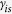 values for the intrinsic (I_2_) and twin-like (T_2_) faults are even negative, implying that the I_2_ (…*ABABABCACACA*…) and T_2_ (…*ABABABCBABAB*…) can form extremely easily. In the basal 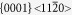 slip system, the 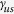 value of *hcp* Au is 12.3% lower than that of *hcp* Mg. This also indicates that the basal stacking faults can form more easily in *hcp* Au than in *hcp* Mg. Once the basal stacking faults generate, the segment of *fcc* crystal will appear, which is regarded as the beginning of *hcp* *→* *fcc* phase transformation. Accordingly, the *hcp* Au is predicted to be unstable. Moreover, the experimental evidence has been validated that *hcp* *→* *fcc* phase transformation of Au can proceed easily, e.g. when the Au sheet is exposed to an electron beam or grows thicker^1^.

In the prismatic 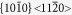 as well as pyramidal 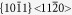 and 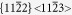 slip systems, the 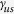 values of *hcp* Au are larger than those of *hcp* Mg by about 15.0%, 22.0% and 42.4% respectively. Therefore, the difficulties are increased in generating non-basal stacking faults for *hcp* Au compared with Mg.

### TB energy

**Figure 4:**
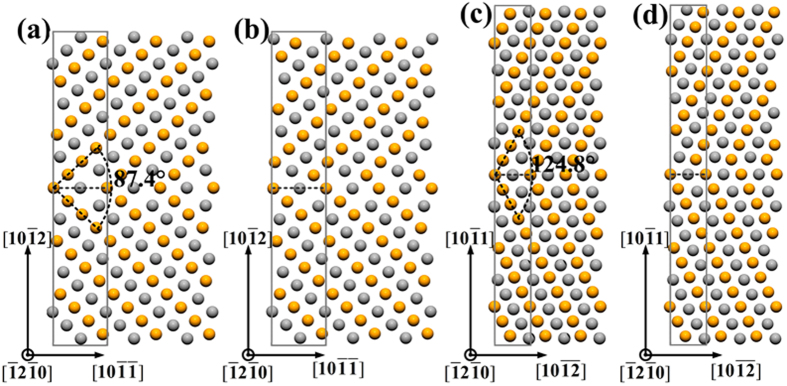
Schematic illustrations of the twin boundaries: (**a**) 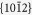 mirror reflection, (**b**) 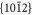 mirror glide, (**c**) 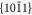 mirror reflection and (**d**) 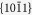 mirror glide. The grey and gold balls represent the two atomic layers along the 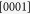 direction conventionally used for the *hcp* structure.

The 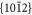 mirror reflection, 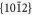 mirror glide, 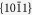 mirror reflection and 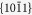 mirror glide twin boundaries were constructed following Wang *et al.*’s work^34^, as illustrated in Fig. 4a–d respectively. The grey and gold balls in supercells represent the two kinds of stacking planes along the 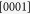 in *hcp* Au. The rotation angle between the *hcp* matrix and the twin is 87.4° and 124.8° for 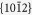 and 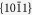 twins, respectively.

The TB energies for *hcp* Au and Mg are summarized in Table 3, and the results of Mg are shown to agree with the data reported by Wang *et al.*^34^. Considering the 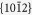 and 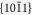 twin boundaries separately, the corresponding TB energies are close in values for mirror reflection and mirror glide-type twins. Therefore, it is predicted that the glide of the interfacial crystal planes have minor effects on the TB energies. Similar results have also been obtained in Wang *et al.*’s work^34^. Moreover, the twin structures with 15 and 17 layers are adopted here to investigate the effects of supercell sizes on the TB energies. For 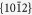 mirror reflection twin boundaries, increasing the supercell size by up to 34 atoms can only decrease the TB energies of *hcp* Au by about 7.8%. For 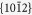 mirror glide, 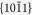 mirror reflection and mirror glide twin boundaries, the TB energies of Au_34_ (17 layers) are slightly larger than those of Au_30_ (15 layers) by less than 3.7%. Accordingly, the calculated TB energies show weak dependence on the adopted supercell size.

The TB energies mainly reflect the stability of twin structures without referring to the nucleation course of twins. By comparison, the TB energies are larger in *hcp* Au than in *hcp* Mg for the four twin types, implying less stability of twin structures in the former case. Moreover, the 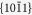 TB energies in *hcp* Au are lower than 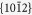 TB energies, which is the same as the situation in Mg.

### Elastic property

For a hexagonal solid, there are five independent elastic constants, namely, C_11_, C_12_, C_13_, C_33_ and C_55_. For a cubic crystal, there are only three independent elastic constants, namely, C_11_, C_12_ and C_44_. The elastic constants for *hcp* Au, Mg and *fcc* Au are calculated and listed in Table 4. Note that our calculated C_ij_ values agree well with the results of Shang *et al.*^37^,^38^, in which the stability of pure elements was successfully discussed in terms of the elasticity. The acceptable tolerance between our results and other simulation^37^,^38^ indicates the accuracy of our work.

Based on the predicted C_ij_’s, the mechanical stability for a given structure can be judged according to Born’s criteria^39^,^40^: 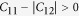, 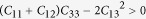 and 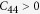 for hexagonal system; and 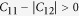, 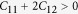 and 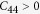 for cubic system.

Note that *hcp* Mg and *fcc* Au, which have stable structures at room temperature, satisfy the above Born criteria for mechanical stability. Meanwhile, the new material *hcp* Au also accords with the Born’s criteria, implying the mechanically metastable. The results of elastic constants confirm the possibility of fabricating pure and stable *hcp* Au under ambient conditions^1^. Although the elastic property demonstrates that *hcp* Au is metastable, the phase transformation from *hcp* to *fcc* is still predicted to proceed easily according to the GSF energies and the surfactant effect, which is also in good agreement with the experimental observation^1^.

Starting from C_ij_’s, the polycrystalline aggregate properties such as bulk modulus (B), shear modulus (G) and Young’s modulus (E) are calculated according to the Voigt^41^ (B_v_ and G_v_), Reuss^42^ (B_r_ and G_r_) and Hill^43^ approximations and indicated in Table 4. Surprisingly, the G of *hcp* Au (19.5 GPa) is comparable to that of *hcp* Mg (21.5 GPa) and *fcc* Au (26.8 GPa). Moreover, the values of B are similar between *hcp* Au (140.2 GPa) and *fcc* Au (137.6 GPa), and are far larger than that for *hcp* Mg (36.4 GPa). Therefore, *hcp* Au possesses potential application prospect, and it is of great significance to *in situ* synthesize the pure *hcp* Au in experiment^1^. Meanwhile, more methods are still in urgent need involving hindering the *hcp* → *fcc* phase transformation and stabilizing *hcp* Au structure.

In summary, we perform systematic first-principles calculations to predict the structural parameters, GSF energies, TB energies and surfactant effect to *hcp* Au, in anticipation of evaluating the structural stability and mechanical properties of this new material. Originating from the self-assembly of alkanethiolate species on *hcp* Au (0001), a slight extension of surface lattice is found, which may be related to the ease of the *hcp* → *fcc* phase transformation. Furthermore, the comparisons are made among *hcp* Au, *fcc* Au and *hcp* Mg. In the basal 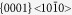 and 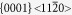 slip systems, the GSF energies (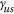) are apparently lower for *hcp* Au than those for *hcp* Mg and even for *fcc* Au. Accordingly, the basal stacking faults with partial *fcc* feature may generate more frequently in *hcp* Au, which facilities the transformation to an *fcc* phase. In the prismatic 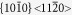 as well as pyramidal 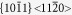 and 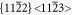 slip systems, the larger 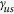 values for *hcp* Au than for *hcp* Mg indicate the increased difficulties in generating non-basal stacking faults in the former case. Moreover, the TB energies of 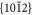 and 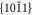 twins are larger in *hcp* Au than in *hcp* Mg, implying less stability of twin boundaries in *hcp* Au. The mechanically metastable of *hcp* Au is proved in terms of Born’s criteria, which verifies the existence of this new phase, however it still shows great tendency to transform to the *fcc* phase because of the easy operation of basal stacking faults and the surfactant effect. The calculated values can serve as the input for the future simulation of the growth process of these planar defects, and contribute to guiding the experiments of fabricating and developing materials with new structure.

## Methods

### Methods and parameters for calculation

The calculation of total energy in this work was performed by the Cambridge Sequential Total Energy Package code (CASTEP)^44^ based on density-functional theory (DFT), in which the Perdew Wang’s^45^ (PW91) version of the generalized gradient approximation (GGA) was employed as exchange correlation functional. The plane-wave cutoff was set to 400 eV. The optimization was performed through the Broyden-Fletcher-Goldfarb-Shanno (BFGS) technique with the convergence tolerances: the energy change less than 5 × 10^−6^ eV/atom, the Hellmann–Feynman force within 0.01 eV/Å and the maximum displacement less than 5 × 10^−4^ Å. The Brillouin zone integration was sampled using dense Monkhorst-Pack^46^ k-point meshes.

The structural properties (equilibrium lattice constants, cohesive energy) of *hcp* Au, Mg and *fcc* Au were evaluated by full optimization on both equilibrium volume and atomic positions. The k-point meshes were samples as: 18 × 18 × 12 for *hcp* Mg and *hcp* Au, and 12 × 12 × 12 for *fcc* Au. The cohesive energy (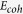) of the pure elements was computed according to Formula (2):


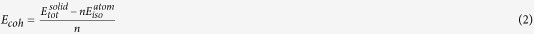


where 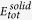 is the total energy of the elements in their ground-state crystal structures, 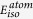 is the energy of isolate atom, and n is the number of atoms in the crystal.

To evaluate the surfactant effect to the lattice constants of *hcp* Au, a 4 × 2 slab (along the 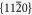 and 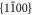 direction) with 9 layers was constructed, separated by a 12 Å vacuum. The plane-wave basis cutoff energy was 400 eV. The 2 × 2 × 1 k-point was utilized according to the Monkhorst-Pack scheme.

### Calculation of the GSF energy

The GSF energy can be obtained by incrementally shifting the upper half crystal along the slip direction and calculating the energy differences per unit area^47^, as shown in Formula (3).


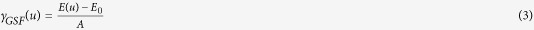


where 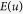 is the total energy of the supercell with the fault vector *u*, 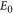 stands for the energy of the perfect lattice, and *A* represents the area of the fault planes. The stacking-fault vector *u* varies from 0.0**b** to 1.0**b** with a step of 0.1**b** for each slip system; hereinto **b** is the corresponding Burgers vector. During geometry optimization, all atoms in supercells were allowed to be relaxed along z-axis, i.e. the direction normal to the slip planes. For the calculation of the GSF energy, the k-point meshes in different systems are listed in Table 2.

A 32-atom *hcp* Au supercell containing 16 layers was constructed to calculate the GSF profiles, as illustrated by Huang *et al.*^1^. Meanwhile, a large vacuum width of 15 Å was added to accommodate the out-of-plane relaxations and to improve the calculation efficiency. The supercells with the same size were also employed for Mg. In the 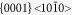 slip system, the intrinsic I_2_ (…*ABABABCACACA*…) stacking fault is generated from the perfect *hcp* structure (…*ABABABABABAB*…), and the twin fault T_2_ (…*ABABABCBABAB*…) can be obtained by the further shear of I_2_.

Moreover, the *fcc* Au slabs consisting of 13 (111) planes were also constructed to calculate the GSF energy in 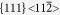 slip system. The intrinsic I (…*ABCABCBCABCA*…) stacking fault can be generated from the perfect *fcc* structure (…*ABCABCABCABC*…), and the two-layer twin fault T (…*ABCABCBABCAB*…) is formed basing on the further shear of I.

### Calculation of the TB energy

The TB energy is depicted as the energy difference between the supercell containing twin boundary and the equivalent in bulk material^34^, as expressed in Formula (4).


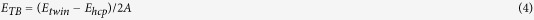


where 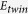 and 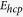 correspond to the total energy of the supercells with and without twin boundaries, and 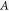 represents the area of twin boundary. Note that full periodic boundary conditions were applied in our DFT calculations for both the twins, and therefore, two twin boundaries exist in each supercell: one is in the middle of supercell and the other is on its top/bottom edge. Accordingly, the 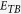 is divided by 2 in Formula (4). The effect of the supercell sizes on the calculated TB energies was studied. The built supercells with different sizes are listed in Table 3, as well as the corresponding k-point meshes.

### Calculation of the elastic constants

The calculation of the elastic constants was performed by the CASTEP code. The Perdew Wang’s^45^ (PW91) version of the generalized gradient approximation (GGA) was employed as exchange correlation functional. The plane-wave cutoff was set to 400 eV. The Brillouin zone was sampled on 18 × 18 × 12 k-point mesh for *hcp* Mg and *hcp* Au and 12 × 12 × 12 for *fcc* Au based on the Monkhorst-Pack scheme^46^. The criteria for the convergence of optimization on atomic internal freedoms were selected as: the energy difference within 1 × 10^−6^ eV/atom, the maximum force within 0.002 eV/Å and the maximum displacement within 1 × 10^−4^ Å.

The elastic stiffness coefficients were determined from a linear fit of the calculated stress as a function of strain^48^. The ground-state structure was strained according to symmetry-dependent strain patterns with varying amplitudes. Subsequently, the stress tensor was computed after a re-optimization of the internal structure parameters, i.e. a geometry optimization with fixed cell parameters. The elastic stiffness coefficients are then the proportionality coefficients relating the applied stress to the computed strain. Two positive and two negative amplitudes were used for each strain component with the value of 0.001 and 0.003 respectively.

The B and G are calculated using the Voigt-Reuss-Hill approximations^43^ for averaging the elastic constants of the single crystal.

For hexagonal system, the Voigt values are expressed as follows^41^:


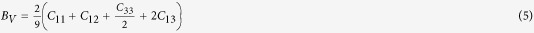



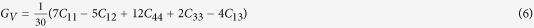


And the Reuss values are calculated according to Formula (7) and (8)^42^:


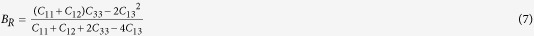



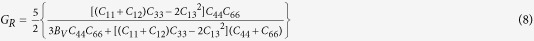


The Hill mean values are obtained by^43^:


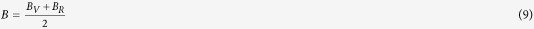



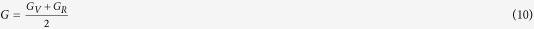



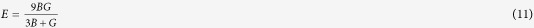


For cubic system, the elastic properties (B, G, E) are calculated as follows^43^:


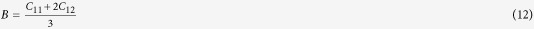



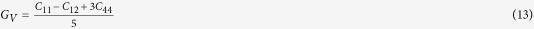



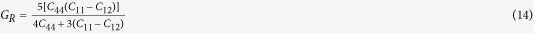



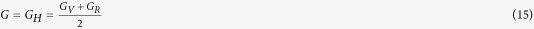



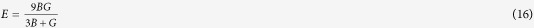


## Author Contributions

C.W. and H.Y.W. conceived and designed the experiments. C.W., F.Q. and T.L.H. performed the model construction and energetic calculations. C.W., H.Y.W. and X.N.X. performed the data analysis. C.W., H.Y.W. and Q.C.J. co-wrote the paper. All authors discussed the results and commented on the manuscript.

## Additional Information

**How to cite this article**: Wang, C. *et al*. Generalized-stacking-fault energy and twin-boundary energy of hexagonal close-packed Au: A first-principles calculation. *Sci. Rep.* **5**, 10213; doi: 10.1038/srep10213 (2015).

## Figures and Tables

**Table 1:** Lattice constants and cohesive energy for *fcc* Au, *hcp* Au and Mg.

**Structure**	**Lattice constant (Å)**		**Cohesive energy(eV/atom)**
	**a**	**c**	
*fcc* Au	4.197	-	−3.2064
*fcc* Au^exp,^ a	4.0786	-	
*fcc* Au^cal,^ b	4.173	-	−3.2026
*hcp* Au	2.952	4.885	−3.2045
*hcp* Au^exp,^ c	2.96	4.84	
*hcp* Au^cal,^ b	2.927	4.903	−3.2018
*hcp* Mg	3.221	5.178	−1.4411
*hcp* Mg^exp,^ d	3.209	5.210	
*hcp* Mg^cal,^ e	3.19	5.17	

^a^Jette, E. R. *et al.*^29^.

^b^Wang, Y. *et al.*^18^.

^c^Huang, X. *et al.*^1^.

^d^Walker, G. B. *et al.*^49^.

^e^Hector Jr., L. G. *et al.*^50^.

**Table 2:** The unstable stacking fault energy ( 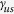 ) and the intrinsic stacking fault energy ( 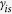 ) for *hcp* Au, Mg and *fcc* Au in different slip systems.

**Slip system**	**Structure**	**k-mesh**	**GSF energy (mJ/m**^**2**^)	
			**Unstable SF (** 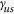 )	**Intrinsic SF (** 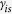 )
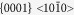	*hcp* Au_32_	5 ×8×1	*γ*_*us*_ (I_2_) 65.9	*γ*_*is*_ (I_2_) −10.5
			*γ*_*us*_ (T_2_) 67.5	*γ*_*is*_ (T_2_) −12.0
	Mg_32_	4 ×8×1	*γ*_*us*_ (I_2_) 95.8, 94.9^b^, 92^c^, 91^f^	*γ*_*is*_ (I_2_) 20.1 21.8^a^, 26.1^b^, 33^c^
			*γ*_*us*_ (T_2_) 120.3 111.2^b^	*γ*_*is*_ (T_2_) 38.2 37.1^b^
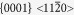	*hcp* Au_32_	5 ×8×1	222.4	–
	Mg_32_	4 ×8×1	253.6, 276^f^	–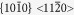	*hcp* Au_32_	8 ×10×1	248.5	–
	Mg_32_	8 ×10×1	216.1, 231^f^	–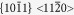	*hcp* Au_32_	8 ×10×1	390.7	–
	Mg_32_	8 ×8×1	320.2 310^d^, 343^f^	–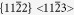	*hcp* Au_32_	8 ×10×1	640.1	309.0
	Mg_32_	8 ×8×1	449.5 485^d^	228.0 236^d^
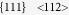	*fcc* Au_13_	10 ×17 ×1	*γ*_*us*_ (I) 83.6 94^e^	*γ*_*is*_ (I) 24.3 27^e^
			*γ*_*us*_ (T) 96.0	*γ*_*is*_ (T) 22.8

^a^Wang, Y. *et al.*^34^.

^b^Jette, E. R. *et al.*^29^.

^c^Han, J. *et al.*^30^.

^d^Nogaret, T. *et al.*^31^.

^e^Wu, X. Z. *et al.*^32^.

^f^Pei, Z. *et al.*^33^.

**Table 3:** Calculated twin boundary (TB) energy for *hcp* Au and *fcc* Au (mJ/m^2^).

**Twin system**	**Structure**	**k-mesh**	**TB energy (mJ/m**^**2**^)	
			This work	Previous calculation
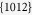 mirror reflection	Au_34_ (17 layers)	8 ×4 ×1	175.2	
	Au_30_ (15 layers)	8 ×4 ×1	190.0	
	Mg_34_ (17 layers)	8 ×3 ×1	120.5	118.8 (40 atoms)^a^
	Mg_30_ (15 layers)	8 ×3 ×1	122.6	122.3 (20 atoms)^a^
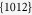 mirror glide	Au_34_ (17 layers)	8 ×4 ×1	189.1	
	Au_30_ (15 layers)	8 ×4 ×1	182.3	
	Mg_34_ (17 layers)	8 ×3 ×1	112.5	120.8 (40 atoms)^a^
	Mg_30_ (15 layers)	8 ×3 ×1	116.8	125.3 (20 atoms)^a^
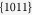 mirror reflection	Au_34_ (17 layers)	9 ×5 ×1	107.3	
	Au_30_ (15 layers)	9 ×5 ×1	106.8	
	Mg_34_ (17 layers)	8 ×4 ×1	80.8	84.2 (40 atoms)^a^
	Mg_30_ (15 layers)	8 ×4 ×1	83.1	85.5 (80 atoms)^a^
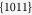 mirror reflection	Au_34_ (17 layers)	9 ×5 ×1	114.3	
	Au_30_ (15 layers)	9 ×5 ×1	110.5	
	Mg_34_ (17 layers)	8 ×4 ×1	80.6	84.2 (40 atoms)^a^
	Mg_30_ (15 layers)	8 ×4 ×1	80.1	81.0 (80 atoms)^a^

^a^Wang, Y. *et al.*^34^.

**Table 4:** Calculated elastic constants for *hcp* Au, Mg and *fcc* Au (GPa).

**Material**	**C**_**11**_	**C**_**12**_	**C**_**13**_	**C**_**33**_	**C**_**44**_	**C**_**66**_	**B**_**r**_	**B**_**v**_	**B**	**G**_**r**_	**G**_**v**_	**G**	**E**
*hcp* Au	174.9	139.8	112.7	182.9	15.1	17.6	140.0	140.3	140.2	18.3	20.7	19.5	56.0
*hcp* Au^cal,^ b	185.7	138.9	112.7	181.3	15.6	-	141.5	142.4	141.9	20.9	23.5	22.2	63.3
*hcp* Mg	63.1	22.2	22.7	66.3	22.6	20.5	36.4	36.4	36.4	21.4	21.5	21.5	53.8
*hcp* Mg^cal,^ a	63.5	24.9	20.0	66.0	19.3	-	-	-	35.83	-	-	18.5	47.4
*fcc* Au	154.4	129.2	-	-	44.1	-	137.6	137.6	137.6	22.1	31.5	26.8	75.4
*fcc* Au^cal,^ b	159.1	136.7	-	-	27.6	-	144.2	144.2	144.2	17.4	21.0	19.2	55.2

^a^Ganeshan, S. *et al.*^37^.

^b^Shang, S. L.*et al.*^38^.
